# Characterization of *TEM1/endosialin *in human and murine brain tumors

**DOI:** 10.1186/1471-2407-9-417

**Published:** 2009-11-30

**Authors:** Eleanor B Carson-Walter, Bethany N Winans, Melissa C Whiteman, Yang Liu, Sally Jarvela, Hannu Haapasalo, Betty M Tyler, David L Huso, Mahlon D Johnson, Kevin A Walter

**Affiliations:** 1 Department of Neurosurgery, The University of Rochester, Rochester, NY, USA; 2 Department of Pathology, Tampere University Hospital, Tampere, Finland; 3 Department of Neurosurgery, The Johns Hopkins Medical Institutions, Baltimore, MD, USA; 4 Department of Molecular and Comparative Pathobiology, The Johns Hopkins Medical Institutions, Baltimore, MD, USA; 5 Department of Pathology, The University of Rochester, Rochester, NY, USA; 6 James P Wilmot Cancer Center, The University of Rochester, Rochester, NY, USA

## Abstract

**Background:**

*TEM1/endosialin *is an emerging microvascular marker of tumor angiogenesis. We characterized the expression pattern of *TEM1/endosialin *in astrocytic and metastatic brain tumors and investigated its role as a therapeutic target in human endothelial cells and mouse xenograft models.

**Methods:**

*In situ *hybridization (ISH), immunohistochemistry (IH) and immunofluorescence (IF) were used to localize *TEM1/endosialin *expression in grade II-IV astrocytomas and metastatic brain tumors on tissue microarrays. Changes in *TEM1/endosialin *expression in response to pro-angiogenic conditions were assessed in human endothelial cells grown *in vitro*. Intracranial U87MG glioblastoma (GBM) xenografts were analyzed in nude *TEM1/endosialin *knockout (KO) and wildtype (WT) mice.

**Results:**

*TEM1/endosialin *was upregulated in primary and metastatic human brain tumors, where it localized primarily to the tumor vasculature and a subset of tumor stromal cells. Analysis of 275 arrayed grade II-IV astrocytomas demonstrated *TEM1/endosialin *expression in 79% of tumors. Robust *TEM1/endosialin *expression occurred in 31% of glioblastomas (grade IV astroctyomas). *TEM1/endosialin *expression was inversely correlated with patient age. TEM1/endosialin showed limited co-localization with CD31, αSMA and fibronectin in clinical specimens. *In vitro*, *TEM1/endosialin *was upregulated in human endothelial cells cultured in matrigel. Vascular *Tem1/endosialin *was induced in intracranial U87MG GBM xenografts grown in mice. *Tem1/endosialin *KO vs WT mice demonstrated equivalent survival and tumor growth when implanted with intracranial GBM xenografts, although *Tem1/endosialin *KO tumors were significantly more vascular than the WT counterparts.

**Conclusion:**

*TEM1/endosialin *was induced in the vasculature of high-grade brain tumors where its expression was inversely correlated with patient age. Although lack of *TEM1/endosialin *did not suppress growth of intracranial GBM xenografts, it did increase tumor vascularity. The cellular localization of *TEM1/endosialin *and its expression profile in primary and metastatic brain tumors support efforts to therapeutically target this protein, potentially via antibody mediated drug delivery strategies.

## Background

Despite advances in neurosurgical techniques, chemotherapeutic regimens and radiotherapy protocols, the prognosis for patients suffering from malignant astrocytoma remains bleak. Novel treatment approaches are required which attack the unique molecular and biological features that contribute to the growth of these tumors. A particularly promising target is the abnormal tumor vasculature, which represents one of the defining characteristics of the most malignant and common astrocytoma, glioblastoma multiforme (GBM). We previously compared the gene expression profile of endothelial cells isolated from freshly resected GBMs with that of endothelium isolated from non-neoplastic temporal lobe [1]. These experiments and subsequent studies identified 21 genes that met the statistical requirements as putative glioma endothelial markers (GEMS). One especially promising candidate was *TEM1/endosialin*.

*TEM1 *was identified in the tumor endothelium of human colon carcinoma [2]. Soon after, it was recognized that *TEM1 *encoded endosialin, which corresponded to the tumor vascular endothelial antigen recognized by the FB5 antibody [3,4]. Further analyses revealed selective *TEM1/endosialin *expression in tumor endothelium, pericytes and a subset of fibroblast-like cells of tumor stroma in breast carcinoma, anaplastic astrocytoma and GBM [5-10]. *TEM1/endosialin *encodes a transmembrane glycoprotein and putative membrane receptor. Recent evidence suggests it may interact with extracellular matrix components including collagen I, collagen IV and fibronection, as well as Mac-2 BP/90K in promoting vascular migration and invasion [11,12]. *Tem1/endosialin *knockout (KO) mice are fertile and appear to develop normally. However, when human HCT116 colon carcinoma cells were implanted orthotopically onto the serosal surface of the large intestine of nude *Tem1/endosialin *KO mice, both tumor take and growth were inhibited while the number of tumor microvessels increased [13]. The selective induction of *TEM1/endosialin *in malignant gliomas, its cell surface bioavailability and the evidence that lack of *TEM1/endosialin *can disrupt tumor growth and vascular differentiation in a xenograft model combine to make it a potential target for molecular therapy.

We sought to expand these preliminary findings by examining the specificity of *TEM1/endosialin *expression in a larger collection of primary and secondary brain tumors. We also wished to determine whether *TEM1/endosialin *induction in brain tumors was conserved in a commonly used U87MG intracranial xenograft model. Finally, we wished to determine whether *TEM1/endosialin *expression was required for brain tumor growth *in vivo*. We confirm that *TEM1/endosialin *is induced, primarily in the vascular compartment, in a wide range of both low-grade and high-grade cerebral neoplasms and is inversely correlated with patient age. TEM1/endosialin shows limited co-localization with fibronectin, a putative binding partner, in malignant brain tumors. *In vitro*, *TEM1/endosialin *expression can be stimulated in human microvascular endothelial cells grown in matrigel. Finally, we show that *Tem1/endosialin *upregulation occurs in the tumor vasculature of intracranial GBM xenografts and absence of *Tem1/endosialin *is associated with increased numbers of microvessels within tumors explanted from *Tem1/endosialin *KO mice, supporting a role for *TEM1/endosialin *in the migration and maturation of the brain tumor vasculature. However, despite its conserved expression pattern in the tumor cerebrovasculature, we find that *Tem1/endosialin *is not required for intracranial tumor growth in *Tem1/endosialin *KO mice.

## Methods

### Clinical specimens

Clinical specimens were provided by the Brain Tumor Bank of the University of Pittsburgh as approved by the University of Pittsburgh Institutional Review Board and the Neurosurgical Brain Tissue Bank of the University of Rochester as approved by the University of Rochester Research Subjects Review Board. All samples were deidentified according to HIPAA regulations prior to being provided for this research. Astrocytoma array materials were obtained from surgical patients at Tampere University Hospital, Tampere, Finland, in 1983-2002, in accordance with the Research Ethics Committee of Tampere University Hospital.

### RNA isolation and reverse transcription

RNA isolations and reverse transcription was performed as previously described [14]. In brief, total RNA was isolated from 10-30 mg of snap frozen, homogenized tissue or approximately 10^6 ^cultured cells using the Qiagen RNeasy Mini Kit (Qiagen, Valencia, CA) according to manufacturer's protocol. cDNA was generated using the SuperScript First-Strand Synthesis System for RT-PCR (Invitrogen, Carlsbad, CA) according to manufacturer's protocol.

### PCR amplification

All PCR primers were generated by IDT (Integrated DNA Technologies, Coralville, IA). Sequences available upon request. PCR reactions were performed using Platinum Taq Polymerase (Invitrogen) as previously described [14]. Reactions were cycled in a PTC-200 DNA Engine thermalcycler (MJ Research, Waltham, MA). Primers for vWF were used for normalization.

### Quantitative RT-PCR amplification

qRT-PCR was carried out on triplicate samples for 40 cycles of 10 s at 95°C, 30 s at 60°C after an initial incubation at 95°C for 3 min in an Chromo4 thermal cycler (Bio-Rad, Hercules, CA). Reaction conditions were 1× iQ SYBR Green super mix (Bio-Rad), 400 nM forward and reverse primers, and 2.0 μl cDNA in a total reaction volume of 25 μl. Amplification of vWF was performed for each cDNA in triplicate for normalization of vascular RNA content. Primer sequences were: TEM1 (F)5'-ctacgttggtggcttcgagt-3'; TEM1(R)5'-caggcctcgtcttcatcttc-3'; vWF(F)5'-cacatcgtgacctttgatgg-3'; vWF(R)5'-gtgcttcacctcgatggatt-3'. Threshold cycle number (*Ct*) of amplification in each sample was determined by Bio-Rad software. Relative mRNA abundance was calculated as the *Ct *for amplification of *TEM1/endosialin *minus average *Ct *for *vWF*, expressed as a power of 2, *i.e*. 2^-ΔCt^. Three individual *TEM1/endosialin *values were averaged to obtain mean ± SD. Statistical significance was determined using a Wilcoxon rank sum test.

### In situ hybridization

Protocol was modified from [14]. Briefly, probe templates blanketing the target cDNA were generated by PCR amplification with incorporation of a T7 promoter into the antisense primer. *In vitro *transcription was performed using the DIG RNA Labeling Kit (SP6/T7) and T7 RNA polymerase (Roche, Indianapolis, IN) according to manufacturer's protocol. Sections were deparaffinized in xylenes and rehydrated in graded ethanols. DIG-labeled RNA probe cocktails were diluted to 200 ng/ml in RNA hybridization solution (EMD Bioscience, Madison, WI) and hybridized to slides overnight at 55°C. Slides were treated with RNase A/T1 (Ambion, Austin, TX) and stringently washed. Slides were blocked with normal rabbit IgG (Dako) diluted 1:20 in buffer (100 mM Tris-HCl, pH 7.5, 150 mM NaCl, 1% casein). For detection, sections were incubated with rabbit HRP-anti-DIG primary (Dako) diluted 1:150 in blocking buffer. Signal was amplified by incubating each slide with one drop of biotinyl-tyramide (Dako). Slides were washed then incubated in goat HRP-anti-biotin secondary (1:100, Vector Laboratories, Burlingame, CA) in blocking buffer. Amplification with biotinyl-tyramide was repeated. Sections were incubated with goat AP-anti-biotin tertiary (1:100, Vector) in blocking buffer and washed. Signal was detected by staining with Fast Red (Sigma-Aldrich, St. Louis, MO) followed by counterstaining with Probe/Hematoxylin counterstain (Biomeda, Foster City, CA). Slides were mounted with Crystal/Mount (Biomeda). Images were captured with an Olympus BH-2 microscope using Spot Insight Color model 3.2.0 camera and Spot Advanced software (Diagnostic Instruments, Sterling Heights, MI).

### Antibodies

TEM1/33, an affinity purified rabbit polyclonal antibody to human TEM1/endosialin, was raised against the peptide RLGFRPAEDDPHRCVDT (Quality Controlled Biochemicals, Hopkinton, MA). Additional goat anti-TEM1/endosialin antibodies sc-48097 and sc-48098 were from Santa Cruz Biotechnology (Santa Cruz, CA). Rabbit-anti-vWF (ab6994) was from Abcam (Cambridge, MA). Goat anti-rabbit-HRP secondary antibody was from Jackson ImmunoResearch Laboratories (West Grove, PA). For immunofluorescence, mouse anti-alpha smooth muscle actin (αSMA) (Sigma), mouse anti-CD31 (Zymed, San Francisco, CA) and mouse anti-fibronectin (FN) (Sigma) were used. Secondary antibodies for immunofluorescence included anti-rabbit Alexa Fluor 568 (red) and anti-goat Alexa Fluor 488 (green) (Invitrogen).

### Immunohistochemistry

Sections were deparaffinized in xylenes and rehydrated in graded ethanols. Slides were washed in 1× PBS and then quenched with 0.3% H_2_0_2 _in 0.3% normal serum/1× PBS for 5 minutes. Slides were washed in 1× PBS and blocked with 4% normal goat serum 1×PBS. Sections were exposed to rabbit polyclonal anti-TEM1 (1:50) or anti-vWF (1:500) at 4°C overnight. Sections were washed with 1× PBS and exposed for 1 hour to goat-anti-rabbit secondary, diluted 1:300. Sections were washed in 1× PBS then exposed to Vectastain-Elite ABC (Vector) for 30 minutes. Sections were washed in 1× PBS and incubated with NovaRed substrate (Vector). Slides were rinsed with distilled water and counterstained with Mayer's Hematoxylin (Biomeda, Foster City, CA). Sections were rinsed with distilled water, exposed to 0.08% NH_4_OH for 3 minutes, rinsed with distilled water and mounted with Crystal/Mount. Images were captured as described above.

### Astrocytoma array

Tumor specimens were fixed in 4% phosphate-buffered formaldehyde and processed into paraffin embedded tissue microarrays as previously described [15]. 275 samples were included in the analyses: 39 grade II, 41 grade III, and 195 grade IV. Patient age ranged from 3 to 80 years (mean ± SD: 50.4 ± 15.2). *TEM1/endosialin *expression was assessed by *in situ *hybridization as described above. Statistical analyses, including Mann-Whitney and Kruskal-Wallis tests to correlate expression to age, were performed using SPSS 11.0 for Windows (SPSS, Chicago, IL).

### Immunofluorescence

Slides were deparaffinzed in xylenes and rehydrated in graded ethanols and rinsed in dH_2_O as described above. For TEM1/CD31, slides were boiled for 15 minutes in citrate buffer (Zymed), cooled for 20 minutes, then washed in PBS for 5 minutes. TEM1/αSMA staining did not require antigen retrieval. For TEM1/FN staining, slides were incubated with 0.1% proteinase K at 37°C for 15 minutes. Slides were washed in PBS and the blocked with 5% normal donkey serum containing 3% BSA in PBS for 30 minutes. Slides were incubated with primary antibodies diluted 1:10 (TEM1/33), 1:20 (CD31), 1:50 (αSMA) or 1:100 (FN) in blocking buffer at 37° for 1 hour. Slides were then rinsed with PBS and incubated with the appropriate secondary in diluted 1:200 in normal host serum for 1 hour at 37°C in the dark. Slides were rinsed in PBS before the addition of ToPro3 nuclear stain (Invitrogen) and then mounted with Prolong Gold mounting media (Invitrogen). Confocal images were captured using Zeiss LSM 510 Axioskop 2 microscope (Zeiss Microimaging, Thornwood, NY) and analyzed with Zen 2007 software (Zeiss).

### Cell lines and culture conditions

Human primary brain microvascular endothelial cells (HPBMEC) (Cell Systems, Kirkland, WA) were maintained in CSC-Complete media (Cell Systems). Human dermal microvascular endothelial cells (HMVEC) (Lonza, Walkersville, MD) were maintained in EBM/EGM-2MV growth media with 1× growth supplements (hEGF, hydrocortisone, GA-1000, FBS, VEGF, hFGF-B, R^3^-IGF-1, and ascorbic acid) (Cambrex). U87MG tumor cells (ATCC, Manassas, VA) were maintained in Minimum Essential Medium Eagle with Earle's Salts and L-glutamine (Invitrogen, Carlsbad, CA), supplemented with 10% FBS, 0.1 mM MEM non-essential amino acids, 0.1 mM MEM sodium pyruvate, sodium bicarbonate, 1 unit/ml penicillin, and 1 μg/ml streptomycin (Invitrogen). Cells were maintained in a 5% CO_2 _atmosphere at 37°C. Endothelial cells were used at passage 3-7 in all experiments. For growth factor experiments, endothelial cells were cultured in the presence of 25 ng/ml VEGF (Sigma), 10 ng/ml TGFβ(R&D Systems), 100 ng/ml IGF-1 (Upstate, Billerica, MA), 50 ng/ml SDF-1 (Biosource, Carlsbad, CA), 20 ng/ml SCF (Biosource) or 50 ng/ml SF/HGF (Biosource) for 48 hours prior to harvest and RNA isolation. Culture of endothelial cells in matrigel (BD Bioscience, Bedford, MA) was performed as described [14]. Cells were harvested from matrigel using Cell Recovery Solution (BD Bioscience) according to manufacturer's protocol. In brief, the cell/matrigel layer was transferred to a 50 ml tube and 4 ml of Cell Recovery Solution was added. The mixture was incubated on ice to completely dissociate the matrigel and release the cells, which were then collected by centrifugation, washed twice in PBS and used for RNA isolation.

### Intracranial xenografts

U87MG cells were harvested with trypsin (Invitrogen) and resuspended at a concentration of 10^5 ^cells/10 μl antibiotic-free DMEM. Athymic nu/nu mice were purchased from the National Cancer Institute (Frederick, MD) and the nude *Tem1/endosialin *KO and WT mice were derived as previously described [13]. Mice were anesthetized using an intraperitoneal injection of 3 ml/kg xylazine/ketamine and tumor cells were stereotactically injected as previously described [16]. Mice were observed daily and survival time was recorded. Mice were euthanized by barbituate overdose when they exhibited symptoms of tumor burden or distress. Brains were surgically removed, formalin fixed and embedded in paraffin. All procedures were approved by the Institutional Animal Care and Usage Committees of the Johns Hopkins University School of Medicine and the University of Rochester.

### Vessel counts

Tumor sections were immunostained for vWF, as described above. Vessels were counted in 10 non-overlapping, 400× fields (5 fields each from two anatomically distinct tumor regions), in 6 *Tem1/endosialin *WT and 6 *Tem1/endosialin *KO xenograft tumors. Statistical significance was determined by Wilcoxon rank sum test.

## Results

### TEM1/endosialin is upregulated in both primary and secondary brain tumors

**Figure 1:**
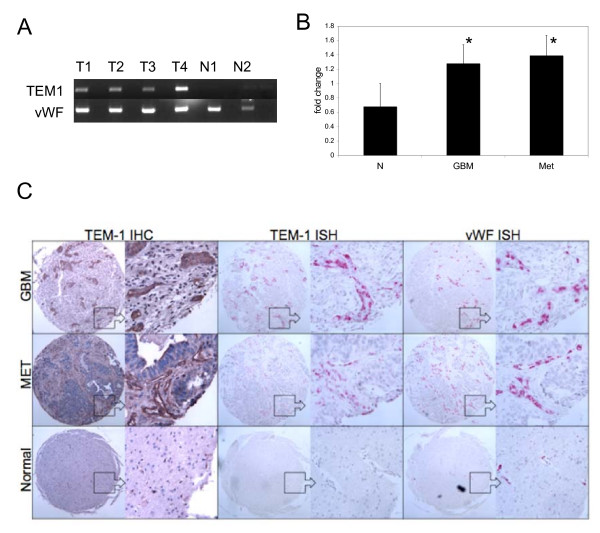
**Localization of *TEM1/endosialin *expression**. A. Representative RT-PCR for *TEM1/endosialin *(TEM1) in whole cell lysates from two GBMs (T1, T2), two metastatic brain tumors (T3, T4) and two non-neoplastic controls (N1, N2). Expression was normalized to von Willebrand Factor (vWF), an endothelial positive control. B. Combined quantitative RT-PCR results for *TEM1/endosialin *in two controls (N), five GBMs (GBM) and five metastatic tumors (Met). Error bars, standard error of the mean. *p = 0.05. C. *TEM1/endosialin *was visualized by immunohistochemistry (IHC) using the TEM1/33 custom antibody or *in situ *hybridization (ISH) in glioblastoma (GBM), metastatic brain tumor (MET) and non-neoplastic brain tissues (Normal). Staining for vWF was used as an endothelial positive control. Original magnification for whole spot, 40×; detail, 100×.

Our previous SAGE data and studies from others indicated that *TEM1/endosialin *was upregulated in the microvasculature of human GBMs and a subset of brain tumors [1,5,7]. In order to confirm that *TEM1/endosialin *induction was common to both primary and secondary brain tumors, we performed quantitative RT-PCR using *TEM1/endosialin *specific primers on mRNA isolated from frozen clinical specimens of 2 non-neoplastic brain tissues and 5 GBMs, as well as 5 specimens of metastatic lung adenocarcinoma resected from brain. Reactions were normalized to vWF in order to account for the increased vascularity of the tumor specimens. Representative standard RT-PCR data are shown in Fig 1A. Quantitative RT-PCR results confirmed that upregulation of *TEM1/endosialin *is detectable in whole tissue from both primary and secondary brain tumor samples as compared to non-neoplastic controls and support a role for *TEM1/endosialin *as an angiogenic marker for CNS neoplasia (Fig 1B).

We next used *in situ *hybridization and immunohistochemistry to localize expression of *TEM1/endosialin *mRNA and protein (Fig 1C) using an independent array of 7 non-neoplastic (reactive) brain tissue specimens, 4 pilocytic astrocytomas, 7 metastatic adenocarcinomas (3 lung adenocarcinomas, 1 lung adenosquamous carcinoma, 1 non-small cell lung carcinoma, 1 esophageal adenocarcinoma and 1 colon adenocarcinoma) and 9 GBMs. *TEM1/endosialin *expression was detected in the vasculature of both GBMs and metastatic brain tumors, as compared to the endothelial control marker, vWF. In GBMs, 3/9 tumors or approximately 33% of the tissues demonstrated a robust staining pattern for *TEM1/endosialin *(nearly 100% of vessels positive), while another 2/9 tumors showed moderate expression (approximately 50% of vessels positive) (Table 1). Similarly, 2/7 metastatic tumors (28%) showed robust staining for *TEM1/endosialin *while another 2/7 (28%) demonstrated a moderate staining pattern. *TEM1/endosialin *expression was occasionally noted in a subset of tumor stromal cells. Little to no staining was detected in reactive (non-neoplastic) brain tissue. Thus, high-grade primary and secondary brain tumors show similar expression profiles for *TEM1/endosialin*. These numbers are consistent with previous work [7], and suggest that the role of *TEM1/endosialin *is critical for vessel development in the brain, regardless of the tumor tissue of origin. Interestingly, 1 of 4 pilocytic astrocytoma specimens showed robust *TEM1/endosialin *expression while the remaining 3 had little to no *TEM1/endosialin *reactivity. Pilocytic astrocytomas are low-grade astrocytomas, but can be highly angiogenic and often demonstrate enhancement and blood-brain barrier disruption on MRI scans.

**Table 1:** *TEM1/endosialin *and fibronectin expression levels in brain tissue microarray.

Tissue	Sample	TEM1	FN
Control	1	-	+
	2	-	+
	3	-	+
	4	-	++
	5	+/-	+
	6	+	+
	7	+/-	++
			
PA	1	+++	+++
	2	+	+
	3	+	+
	4	-	++
			
Met	1	+++	+++
	2	+	+++
	3	++	++
	4	+	++
	5	+++	++
	6	++	++
	7	+	++
			
GBM	1	+	+
	2	++	++
	3	+/++	++
	4	+	+/++
	5	+++	+
	6	++	+
	7	+	+
	8	+++	+++
	9	+++	++

*NOTE*: Non-neoplastic control tissue (Control), pilocytic astrocytoma (PA), metastatic brain tumors (Mets) and glioblastoma (GBM). TEM1/33 and mouse anti-fibronectin antibodies were used. (-) no staining; (+) weak staining or 10% vessels; (++) moderate staining or approximately 50% of vessels; and (+++) strong staining or nearly 100% of vessels.

As astrocytomas account for more than half of primary CNS malignancies, we expanded our examination to a larger group of primary astrocytic brain tumors in order to look for associations between *TEM1/endosialin *expression and clinically relevant pathologic features. We used *in situ *hybridization to probe expression of 275 arrayed grade II-grade IV astrocytic tumors. The results are summarized in Table 2. Overall, *TEM1/endosialin *expression was detected in 79% of tumor samples (Table 2). When we focused on the grade IV astrocytomas (GBMs) alone, which constitute the most aggressive and lethal subtype, 31% of the tumors demonstrated moderate to strong staining for *TEM1/endosialin *(Table 2). These data confirm that *TEM1/endosialin *is widely expressed in astrocytic tumors. *TEM1/endosialin *expression in grade II-IV astrocytomas demonstrated a statistically significant inverse correlation with patient age (p = 0.004) (Table 2), although it showed no correlation with other common measures such as tumor proliferation index (MIB-1) or apoptotic rate.

**Table 2:** Expression of *TEM1/endosialin *in grade II-IV astrocytoma microarray.

**Tumor**	**Negative**	**Positive**	**Total**
Grade II	7	32	39
Grade III	8	33	41
GBM	42	153	195
Total	57	218	275
			
**Tumor**	**Negative**	**Weak**	**Mod/Strong**
GBM	42	91	62
			
	**TEM1 Neg/Weak** **(N = 156)**	**TEM1 Mod/Strong (N = 93)**	
**Age (years)**			
Mean	54.8	49.1	
Median	57.2	50.5	
SD	15.2	15.5	

*NOTE*: Positive staining as measured by ISH was defined as: weak or 10% of vessels; moderate or approximately 50% of vessels; and strong or nearly 100% of vessels. *TEM1/endosialin *expression inversely correlated with patient age in grade II-IV astrocytomas for which age data was available, p = 0.004, Mann-Whitney test. SD: standard deviation.

### Co-localization of TEM1/endosialin and fibronectin

**Figure 2:**
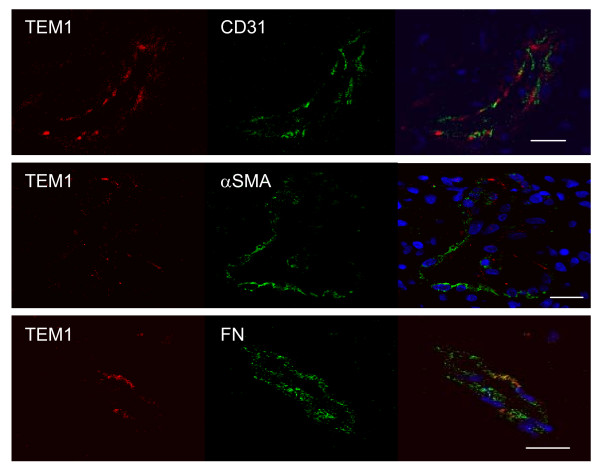
**Immunofluorescence for TEM1/endosialin: co-localization in tumor specimens**. TEM1 and CD31 (top panel), αSMA (middle panel) and fibronectin (FN, bottom panel) co-localize to microvessels in human GBMs, but demonstrate limited cellular overlap. Anti-TEM1 (TEM1/33), red. Anti-CD31, anti-αSMA and anti-FN, green. Nuclei, blue. Scale bar, 20 μm.

Fibronectin (FN), collagen I and collagen IV were recently identified as potential binding partners for TEM1/endosialin [11]. We therefore stained the same GBM/metastatic brain tumor tissue array for FN to see if there was a correlation between TEM1/endosialin and FN expression patterns. TEM1/endosialin and FN were co-upregulated in some tumors, although the correlation was not consistent (Table 1). A similar pattern of upregulation was noted for collagen I and collagen IV (YL, unpublished data). Using immunofluorescence, we detected limited co-localization of TEM1/endosialin and FN in GBM and metastatic brain tumor sections, suggesting that physiologic interactions between TEM1/endosialin and FN may occur, but that such interactions are not extensive in clinical settings of CNS neoplasia (Fig 2 and Additional file 1). Similar results were obtained using a custom synthesized, affinity purified anti-TEM1 antibody or commercial antibodies, targeting 3 different epitopes within the protein.

### Regulation of TEM1/endosialin expression in vitro

*TEM1/endosialin *was originally characterized as an endothelial cell marker, although recent studies have provided evidence for its expression in endothelial progenitor cells, mesenchymal stem cells, tumor pericytes, all of which contribute to the tumor vasculature, and a subpopulation of tumor stromal cells [8,10,17-20]. Co-immunofluorescence for TEM1/endosialin and the pericyte marker alpha-smooth muscle actin (αSMA) in the microvasculature of our GBMs and metastatic brain tumors revealed minimal overlap of the two proteins at the cellular level, although the markers did routinely track along the same vessels (Fig 2). Co-immunofluorescence for TEM1/endosialin and CD31, an endothelial marker, also demonstrated limited co-localization of the two proteins, although again both proteins were found on shared vessels (Fig 2). Our results differ in degree slightly from those of Simonavicius, *et al*. [5], but this may be due to differences in antibodies, tissue fixation conditions (Rouleau *et al*. experienced significantly different TEM1/endosialin staining results from FFPE versus frozen tumor tissues [21]) or clinical tumor heterogeneity.

**Figure 3:**
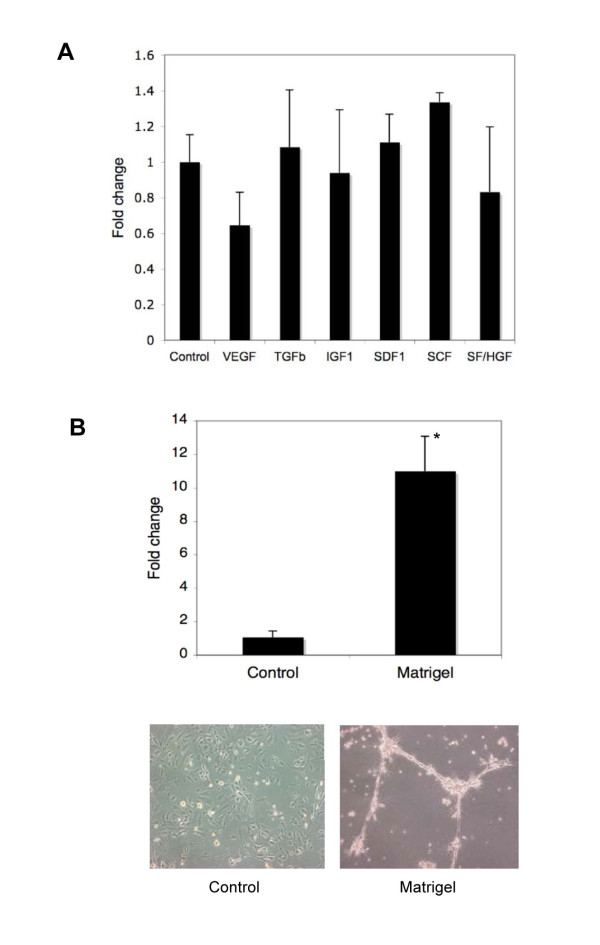
**Expression of *TEM1/endosialin *by cultured endothelial cells**. A. Expression of *TEM1/endosialin *after treatment of endothelial cells with pro-angiogenic growth factors. Cells were treated for 48 hours (see Methods) and analyzed by quantitative RT-PCR. Error bars, standard error of the mean. No significant change in *TEM1/endosialin *was detected. B. Induction of *TEM1/endosialin *after growth of endothelial cells in matrigel. Cells were grown on plastic (Control) or in matrigel (Matrigel) for 6 hours then analyzed by quantitative RT-PCR. Error bars, standard error of the mean. *p = 0.05. After 6 hours on matrigel and prior to harvest, HMVEC cells demonstrate branching tubulogenesis (lower right) while cells grown on plastic do not (lower left).

We wished to determine factors that may induce *TEM1/endosialin *in the endothelium of human brain tumors in order to further clarify the role of this protein. Little or no *Tem1/endosialin *induction was detected in human microvascular endothelial cells (HMVECs) or human brain microvascular endothelial cells (HBMECs) after exposure to pro-angiogenic growth factors, including vascular endothelial growth factor (VEGF), transforming growth factor beta (TGF-β), insulin-like growth factor 1 (IGF1), stem cell factor (SCF), stromal cell-derived factor 1 (SDF-1) and scatter factor/hepatocyte growth factor (SF/HGF) (Fig 3A). Likewise, culturing primary endothelial cells under hypoxic conditions (2% oxygen for 24 hours) did not induce *TEM1/endosialin *expression (YL, unpublished data). Since it has been proposed that *TEM1/endosialin *may interact with proteins within the extracellular matrix as well as tumor stromal cells to promote vascular invasion and migration [11], we examined the effects of growth in matrigel on *TEM1/endosialin *expression in HMVEC cells. After 6 hours in matrigel, cells were harvested and *TEM1/endosialin *expression was assessed by quantitative RT-PCR using human specific primers. HMVEC cells cultured in matrigel induced *TEM1/endosialin *ten-fold compared to HMVEC cells cultured on plastic (Fig 3B). No expression was detected in reverse transcriptase negative samples or in matrigel-only control isolates. This suggests that microvascular endothelial cells can induce *TEM1/endosialin *when exposed to a complex extracellular environment.

### TEM1/endosialin is induced in intracranial GBM xenografts

**Figure 4:**
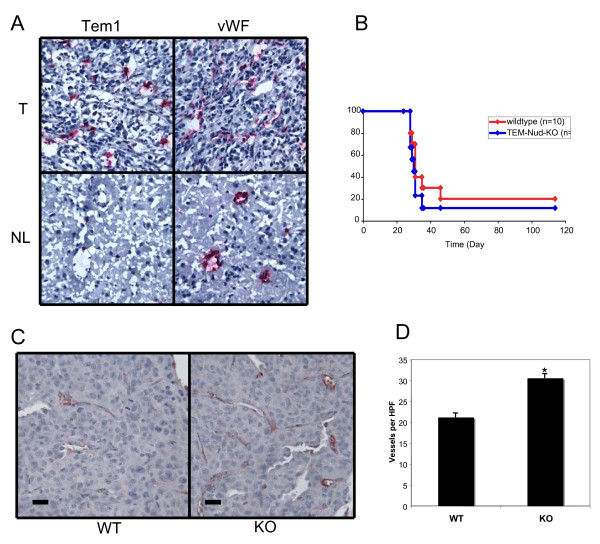
***Tem1/endosialin *expression in mouse brain tumor models**. A. Induction of *Tem1/endosialin *in intracranial U87MG xenografts grown in nude mice. ISH with mouse specific riboprobes detected *Tem1/endosialin *(Tem1) in tumor (T) but not normal mouse brain (NL). Probes for mouse vWF were used as a positive endothelial control (vWF). Original magnification, 200×. B. Survival of nude *TEM1/endosialin *WT and KO mice after intracranial injection with 10^5 ^U87MG glioblastoma cells. Animals sacrificed after 100 days showed no evidence of tumor take. C. Tumor vessels stained for vWF in intracranial xenografts from nude *TEM1/endosialin *WT or KO mice. Size bar, 50 μm. D. Comparison of vessel numbers from nude *TEM1/endosialin *WT and KO tumors. Ten non-overlapping high power fields were counted per animal. Error bars, standard error of the mean. *p = 0.001.

Previous studies demonstrated robust *TEM1/endosialin *induction in B16 melanoma and HCT116 colon carcinoma xenografts grown in mice, while very low levels of *TEM1/endosialin *expression were reported in normal adult mouse brain [9]. We therefore sought to determine whether induction of *TEM1/endosialin *was conserved in an intracranial GBM xenograft model. Human U87MG glioblastoma cells were implanted intracranially into athymic nude mice. *In situ *hybridization using mouse specific riboprobes was used to measure mouse *TEM1/endosialin *expression in the subsequently harvested tumors. The results demonstrated mouse *TEM1/endosialin *induction in the vasculature of the tumor, but not in the surrounding normal mouse brain (Fig 4A). Staining for *TEM1/endosialin *was often concentrated near the margin of the tumor, although this observation did not reach statistical significance, a characteristic noted in human brain tumors as well (BNW, unpublished data).

### Tem1/endosialin expression is not required for brain tumor growth in vivo

In order to investigate whether induction of *Tem1/endosialin *is required for tumorigenesis and/or angiogenesis in brain tumors, we injected U87MG human GBM cells intracranially into nude *Tem1/endosialin *KO and WT mice. No significant difference in survival time between the *Tem1/endosialin *KO and WT animals was detected (Fig 4B). Neither was there a detectable difference in tumor take or volume. We were able to detect *Tem1/endosialin *in the vasculature of the WT, but not the KO, tumors, confirming the genotype of the mice.

Since *TEM1/endosialin *expression has been noted in tumor stromal cells, as well as vascular and perivascular cells, we screened the *Tem1/endosialin *KO and WT tumors for expression of human *TEM1/endosialin*. We did not detect appreciable *TEM1/endosialin *staining by *in situ *hybridization in the tumor stroma of either the knockout or wildtype xenografts. This suggests that growth of the intracranial xenografts was not supplemented by *TEM1/endosialin *expression from the U87MG tumor cells themselves.

Nanda, *et al*., reported increased numbers of microvessels in xenografted colon carcinomas grown in the intestine of nude *Tem1/endosialin *knockout mice as compared to their wildtype counterparts [13]. We therefore examined the intracranial U87MG xenografts for a similar phenotype. The tumors from the *Tem1/endosialin *KO animals had approximately 40% more vessels than the tumors grown in the wildtype mice (Fig. 4C, D). This suggests that although GBM xenografts grew normally in the *Tem1/endosialin *knockout animals, tumor angiogenesis was nevertheless altered. Staining with antibodies to αSMA did not demonstrate any difference in pericyte coverage between the WT and KO tumor vessels (MCW, unpublished data). So while *Tem1/endosialin *is not required for overall growth of this intracranial brain tumor model, it does appear to be required for normal development of the tumor cerebrovasculature, although the mechanism for this remains unclear.

## Discussion

*TEM1/endosialin/CD248 *encodes a 165 kD single-pass transmembrane glycoprotein. It is a C-lectin type receptor with homology to thrombomodulin. Several studies identified *TEM1/endosialin *as a marker of tumor endothelium and isolated activated fibroblasts within the tumor stroma [7,9]. Little to no expression of *TEM1/endosialin *was noted in normal adult tissues. Alternate studies have argued that TEM1/endosialin in the tumor vasculature is expressed by vessel-associated pericytes [8,10,19]. Two additional reports identified TEM1/endosialin in populations of VEGFR2^+^/CD31^+^/CD45^-^/VE-cadherin^+ ^endothelial precursor cells derived from CD133^+^/CD34^+ ^cells or in CD45^+^/VE-cadherin^+ ^"vascular leukocytes" [20,22]. Both cell types have been proposed to contribute to tumor vascularization, although the degree to which either one incorporates into tumor vessels remains controversial. Human umbilical vein endothelial cells immortalized with SV40 large/small T-antigen, a model of proliferating neovascular endothelial cells, also express *TEM1/endosialin *[23]. We argue that *TEM1/endosialin *is expressed to varying degrees by tumor endothelial cells, pericytes and stromal fibroblasts. In normal blood vessels, the pericytes bind tightly to endothelial cells and influence migration, stabilization and maturation of the endothelium. In the cerebrovasculature, pericytes are additionally critical for maintenance of the differentiated blood-brain barrier (BBB) and failure of the BBB in brain tumors is associated with disruption and loss of pericytes lining endothelial cells. Pericytes are of emerging interest as therapeutic targets as vessels may be more sensitive to chemotherapy or radiotherapy when denuded of pericytes. When Bergers, *et al*., targeted both the endothelial cell and pericyte compartments with combinatorial receptor tyrosine kinase inhibitory (RTKI) therapies, they achieved more favorable responses than with either therapy alone [24]. Cao, *et al*., demonstrated that systemic overexpression of Ang2 blocked pericyte recruitment to tumor vessels and led to massive vascular destabilization and tumor suppression [25]. The collective data support the hypothesis that *TEM1/endosialin *has a favorable expression profile for targeting both the endothelial and pericytic portions of the tumor vasculature.

Expression of TEM1/endosialin by a subset of tumor fibroblasts may also influence tumor growth and angiogenesis. Activated tumor fibroblasts contribute to the deposition of fibronectin, collagen I and collagen IV, all of which may bind TEM1/endosialin and guide vessel invasion, migration or differentiation. SDF-1 expression by activated tumor fibroblasts has been shown to drive tumor progression and angiogenesis in breast carcinoma models and has been reported to recruit endothelial progenitor cells to the microvasculature of murine gliomas, where differentiation into both pericytes and endothelium was detected [26,27]. A recent genetic study reported that TGFβ1 treatment of proliferating endothelial cells led to an endothelial-mesenchymal transition, wherein endothelial cells downregulated CD31 and upregulated fibroblast-specific protein-1 (FSP-1). They confirmed the presence of FSP1^+^/CD31^+ ^intermediate cells in tumor models [28]. Further work is necessary to fully determine the complete expression profile of *TEM1/endosialin *and its relationship to these intricately related stromal cell populations.

In the US, primary brain tumors are diagnosed in approximately 40,000 new patients a year. In addition, brain metastases occur in 20-40% of cancer patients, leading to 200,000-400,000 new cases of brain metastases a year. Despite differences in the molecular fingerprints of the tumor cells themselves, it may be possible to identify common changes in the vasculature of these disparate tumor types. Initial studies by ourselves and others reported *TEM1/endosialin *upregulation in human brain tumors [1,5,7]. We wanted to expand these findings in order to better establish the full extent of *TEM1/endosialin *expression in brain tumors. This study confirms expression of *TEM1/endosialin *at the RNA and protein level in astrocytic and metastatic brain tumors, with little to no expression in normal control brain. Expression was primarily limited to the tumor vasculature but occasional stromal cells also stained for *TEM1/endosialin*. Greater than 30% of GBMs displayed robust *TEM1/endosialin *expression, while lesser percentages of lower grade tumors expressed *TEM1/endosialin*. Unfortunately, we did not know whether the individual specimens analyzed were from the tumor center or closer to the tumor margin. Preliminary mouse xenograft data suggested that Tem1/endosialin staining was increased along the tumor margin, but this needs to be further investigated in both clinical and mouse tumors. It will be especially useful to assess *Tem1/endosialin *expression in future experiments using xenografted primary glioma spheroids or glioma stem cells, which grow as a more highly invasive intracranial tumor than the U87 model, although with less pronounced angiogenesis. Interestingly, one pilocytic astrocytoma displayed robust expression of *TEM1/endosialin*. Although pilocytic astrocytomas are considered low-grade tumors, they are highly angiogenic and often enhance on MRI, which is indicative of vascular leakiness, disruption of the blood-brain barrier and often more aggressive disease. A recent study in breast cancer identified an association between *TEM1/endosialin *expression and progressive and/or recurrent disease as well as nodal involvement, all hallmarks of a more aggressive disease state [6]. Thus, TEM1/endosialin may have prognostic value for breast cancer. We did detect a statistically significant (p = 0.004) inverse correlation between *TEM1/endosialin *expression in astrocytomas and patient age, although the implications of this remain unclear. While diagnosis at a younger age can be a predictor of long-term survival for GBM [29], our initial analyses reveal no difference in survival between *TEM1/endosialin *expressing and non-expressing tumors.

Tomkowicz, *et al*. recently identified collagen types I and IV and fibronectin as potential ligands for TEM1/endosialin [11]. They showed that CHO cells engineered to overexpress TEM1/endosialin (CHO-TEM1) exhibited greater adhesion to fibronectin than control CHO cells (CHO-K1). They suggested a model by which TEM1/endosialin is induced in cells associated with endothelium and stromal fibroblasts. The subsequent interactions between TEM1/endosialin expressing cells and fibronectin, collagen I or IV present in the extracellular matrix drive cellular migration required for tumor invasion and angiogenesis. In an independent study, TEM1/endosialin and collagen IV were shown to colocalize in finger-like processes during angiogenic development in the human fetal telencephalon [30]. We had identified collagen I and collagen IV as putative glioma endothelial markers which exhibited a minimum 4-fold induction as compared to normal endothelial cell samples and were induced in both glioma and colon carcinoma endothelium [1]. Therefore, we were curious to determine whether we could detect co-localization of fibronectin with TEM1/endosialin in our primary and secondary tumor samples. We did detect upregulation of both TEM1/endosialin and fibronectin within the same tumor specimens, although only a subset of the samples showed co-localization along the vasculature, supporting a physiologic interaction of these proteins. In addition, Tomkowicz and colleagues showed that the CHO-TEM1 cells, but not CHO-K1 control cells, grew with web-like morphology and formed large clusters when cultured in matrigel. The CHO-TEM1 cells also exhibited enhanced migration through matrigel that was blocked by incubation with a humanized antibody targeted against TEM1/endosialin [11]. Others have shown that incubation with anti-TEM1/endosialin antibodies can block tube formation by endothelial progenitor cells and mesenchymal stem cells [17,20]. Our *in vitro *data showed that human endothelial cells grown in matrigel induce TEM1/endosialin themselves in response to the culture environment. Together, the biochemical and localization data support a functional role for TEM1/endosialin in promoting cell-cell interactions and migration during tumor angiogenesis.

Since the expression profile of *TEM1/endosialin *and existent xenograft data suggested *TEM1/endosialin *might be a viable therapeutic target for brain tumors, we wished to ascertain whether *TEM1/endosialin *induction was conserved in an orthotopic model of GBM. Intracranial U87MG xenografts demonstrated robust upregulation of *Tem1/endosialin *in the tumor vasculature with little to no expression in non-neoplastic brain. Again, staining was predominantly present in the vessels, although we could detect staining in occasional tumor stromal cells. Interestingly, when we implanted U87MG cells engineered to stably overexpress VEGF, a small percentage of tumor cells stained positive for human *TEM1/endosialin*, although there was no significant difference in *Tem1/endosialin *staining in the mouse vessels (BNW, unpublished data).

When we implanted intracranial GBM xenografts into nude *Tem1/endosialin *KO and WT mice, we detected no difference in tumor take or survival. This differs from the colon carcinoma data and suggests that the impact of Tem1/endosialin in the cerebral microenvironment may differ from its role in intestinal tumorigenesis. Nanda, *et al*., reported a significant decrease in tumor take and volume when they implanted colon carcinoma xenografts into the large intestine of nude *Tem1/endosialin *KO mice [13]. However, they did not detect a difference in tumor growth when identical cells were implanted subcutaneously into KO and WT animals. They argued that this emphasized the importance of an anatomically relevant microenvironment for tumor growth and study. This is a similar argument to that proposed by Camphausen, *et al*., who compared the expression profiles of U87MG and U251MG GBM cell lines grown *in vitro *to those from cells grown as subcutaneous or intracranial xenografts [31]. They noted that the expression profiles in tissue culture were significantly different from the subcutaneous profiles, which were, in turn, distinct from the intracranial expression profiles. The disparity between GBM xenograft expression profiles suggested that the orthotopic implantation site can dictate gene expression, presumably due to signaling between the tumor cells and the surrounding stroma. In the current scenario, it would again appear that *TEM1/endosialin *expression levels within distinct anatomical compartments differentially influence tumor growth and pathology. Importantly, the data suggest that inhibition of *TEM1/endosialin *function alone in tumors is insufficient for suppression of brain tumor growth.

Despite unchanged tumor growth and equivalent animal survival between *Tem1/endosialin *WT and KO animals, we did detect an increase in the number of microvessels present in the KO GBM tumors. This strengthens a role for TEM1/endosialin in the maturation of the tumor neo-vasculature [13]. TEM1/endosialin was recently reported to be upregulated by hypoxia via a HIF-2 pathway in a limited number of cell lines, although we could detect no induction in primary human endothelial cells [32]. However, the brain is a highly vascularized site, and it may be that the level of oxygenation is sufficient to promote xenograft growth in the absence of *Tem1/endosialin *induction. It is more likely *TEM1/endosialin *is directly or indirectly required for differentiation or maturation of the vasculature, as opposed to strict proliferation, and that lack of TEM1/endosialin protein leads to the overabundance of vessels demonstrated in the knockout mouse tumors. Further studies are needed to determine if the increased vascularity of the *Tem1/endosialin *KO tumors could render them more sensitive to radiation or chemotherapy, by enhancing the delivery of radiosensitizing oxygen or therapeutic drugs.

## Conclusion

The combined tumor data confirm that TEM1/endosialin contributes to the development of the brain tumor vasculature. Although lack of TEM1/endosialin expression did not inhibit brain tumor growth in our model, the cell surface bioavailability of TEM1/endosialin and its localization to tumor and tumor vasculature components in both primary and metastatic brain tumors, both diseases with limited current treatment options, support efforts to target this protein with alternative approaches, including antibody or antibody-cytotoxin based strategies, such as a recently reported anti-TEM1/endosialin IgG conjugated to saporin, and other efforts in pre-clinical development [11,21,33] or as combination therapy.

## Competing interests

The authors declare that they have no competing interests.

## Authors' contributions

EBC-W designed the studies, contributed ISH and IH experiments and analysis and wrote the manuscript. BNW participated in IH and cell culture experiments and analysis. MCW performed IF experiments and analysis. YL participated in cell culture and gene expression experiments. HH and SJ collected astrocytoma samples, performed microarray experiments and statistical analysis. BMT and DLH performed the animal experiments. MDJ participated in microtissue array construction and pathologic consultation. KAW participated in experimental design, analysis and interpretation and edited the manuscript. All authors have seen and approved the manuscript.

## Pre-publication history

The pre-publication history for this paper can be accessed here:

http://www.biomedcentral.com/1471-2407/9/417/prepub

## Supplementary Material

Additional file 1 **Co-localization of TEM1/endosialin with fibronectin in clinical brain tumor specimens**. Additional immunofluorescence results demonstrating limited co-localization of TEM1/endosialin and fibronectin in GBM using commercial anti-TEM1/endosialin antibodies. Click here for file
