# Supplementary figures and images for: Characterization of TEM1/endosialin in human and murine brain tumors
Source: BMC Cancer. 2009 Nov 30;9:417. doi: 10.1186/1471-2407-9-417 (PMC2793264; doi:10.1186/1471-2407-9-417)

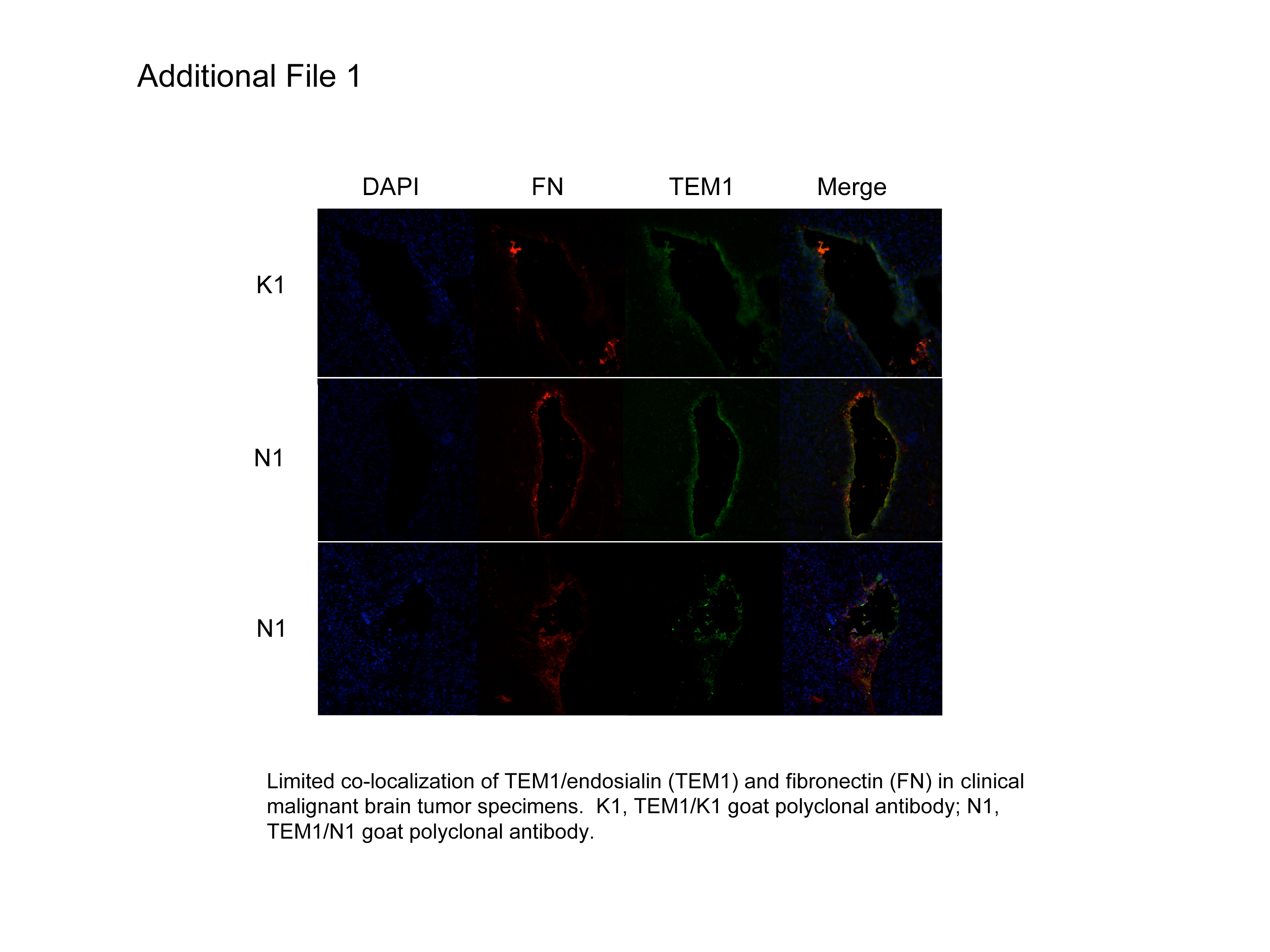

Supplement: Additional file 1 — Co-localization of TEM1/endosialin with fibronectin in clinical brain tumor specimens. Additional immunofluorescence results demonstrating limited co-localization of TEM1/endosialin and fibronectin in GBM using commercial anti-TEM1/endosialin antibodies. [file 1471-2407-9-417-S1.JPEG]
